# Influence of the Cholinergic System on the Immune Response of Teleost Fishes: Potential Model in Biomedical Research

**DOI:** 10.1155/2013/536534

**Published:** 2013-11-13

**Authors:** G. A. Toledo-Ibarra, A. E. Rojas-Mayorquín, M. I. Girón-Pérez

**Affiliations:** ^1^Universidad Autónoma de Nayarit (UAN), Secretaría de Investigación y Posgrado, Laboratorio de Inmunotoxicología, Boulevard Tepic-Xalisco s/n, Cd de la Cultura Amado Nervo, 63190 Tepic, Nayarit, Mexico; ^2^Departamento de Ciencias Ambientales, Instituto de Neurociencias, Centro Universitario de Ciencias Biológicas y Agropecuarias (CUCBA), Universidad de Guadalajara (UdeG), Francisco de Quevedo 180, Col. Arcos Vallarta, 45100 Guadalajara, Jal, Mexico; ^3^Departamento de Investigación Básica, Instituto Nacional de Geriatría (INGER), Periférico Sur No. 2767, Col. San Jerónimo Lídice, Del. Magdalena Contreras, 10200 México, DF, Mexico

## Abstract

Fishes are the phylogenetically oldest vertebrate group, which includes more than one-half of the vertebrates on the planet; additionally, many species have ecological and economic importance. Fish are the first evolved group of organisms with adaptive immune mechanisms; consequently, they are an important link in the evolution of the immune system, thus a potential model for understanding the mechanisms of immunoregulation. Currently, the influence of the neurotransmitter acetylcholine (ACh) on the cells of the immune system is widely studied in mammalian models, which have provided evidence on ACh production by immune cells (the noncholinergic neuronal system); however, these neuroimmunomodulation mechanisms in fish and lower vertebrates are poorly studied. Therefore, the objective of this review paper was to analyze the influence of the cholinergic system on the immune response of teleost fish, which could provide information concerning the possibility of bidirectional communication between the nervous and immune systems in these organisms and provide data for a better understanding of basic issues in neuroimmunology in lower vertebrates, such as bony fishes. Thus, the use of fish as a model in biomedical research may contribute to a better understanding of human diseases and diseases in other animals.

## 1. Immune System in Teleost Fishes

**Figure 1:**
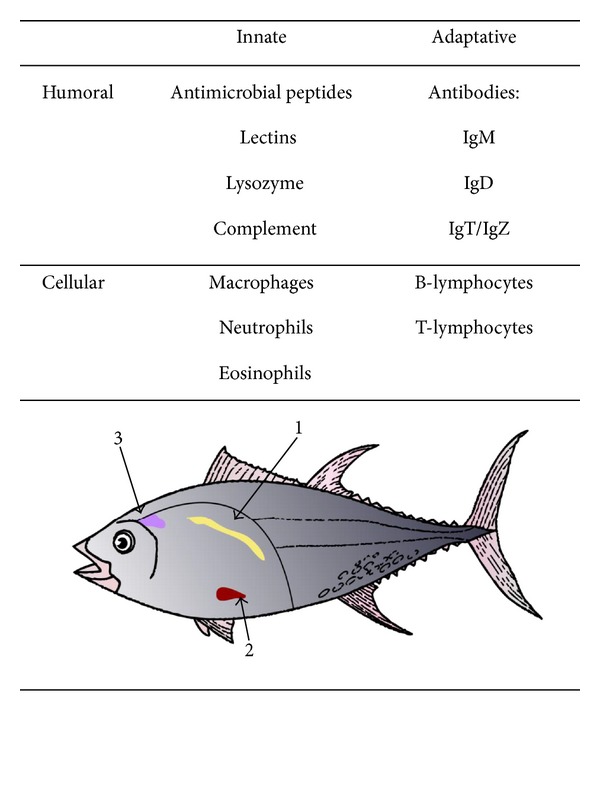
Main humoral, cellular, and anatomical components of the immune system in fishes. Fish lymphoid organs: pronephros (1), spleen (2), and thymus (3).

Fishes are the phylogenetically oldest vertebrate group and appeared >560 million years ago. This group includes >27,000 species, representing more than one half of the vertebrates on the planet. The vast majority of fishes are teleosts (teleostei, possessing a bony skeleton) and some are noted for their ecological and economic importance, whereas other species are widely used as biological models for genomic studies and developmental biology [1, 2]. In addition, because these organisms are the first that present adaptive immune mechanisms (Figure 1), the Big Bang of Immunology [3], the study of the immune system of these organisms is of great relevance because it provides information on the evolution of the immune system in vertebrates, thus supporting the understanding of basic aspects of immunology, therefore the possible treatment of emerging diseases in humans and in other animals [4].

### 1.1. Lymphoid Organs

Fishes, unlike mammals, lack lymph nodes and bone marrow [5]. However, the anterior kidney or pronephros, analog evolutionary of the bone marrow, possesses important hematopoietic functions (precursor hematopoietic cells appear after 96 h as postfertilized (hpf) in mesonephric tubules) and also presents similar functions to those of the adrenal gland of mammals, which is key in connections among the neuroimmune-endocrine systems [6–8]. Additionally, the spleen is the main secondary lymphoid organ in fish and presents a significant number of (IgM^+^ B) lymphocytes, in addition to participating in the induction of adaptive immune responses, and is important for the elimination of immune complexes [4]. Regarding the thymus, this is a bilobed organ localized in the opercular cavity; it is the major site for T-cell development in fish, as well as in mammals, and presents an involution, except that this phenomenon is greatly influenced by hormonal cycles and environmental changes in the latter [9].

### 1.2. Innate Immunity

With respect to the mechanisms of innate immunity, these are important in early defense against pathogen infection [10] and also play an instructor role in the induction of the adaptive response [4]. The innate humoral components that are mainly characterized in fishes are antibacterial peptides, lysozymes, lectins, acute-phase proteins, and molecules of the complement system (Figure 1) [11–15].

The cells of the innate immune system are activated by Pathogen recognition receptors (PRR), an important type of these are the Toll-like receptors (TLR). In fishes, it has been described that the majority of TLR are present in mammals (TLR1, TLR2, TLR 3, TLR4, TLR5, TLR7, TLR8, and TLR9). In addition, in fishes the presence of some TLR that has not yet been identified in mammals (TLR18–TLR23) has been described. Additionally, in channel catfish (*Ictalurus punctatus*), the presence has been identified of TLR25 and TLR26, which are apparently present only in fishes [16, 17]. Finally, there are other TLRs, at least eight more, which have been described in other taxa but not in fishes. 

The cells of the innate immune response mainly characterized in fishes are the macrophages, neutrophils, and eosinophils [16]. Macrophages are phagocytic cells that are important in early antimicrobial responses; it is been suggested that the phagocytic process in fish is more effective than in murine models when both are compared [18]. Additionally, neutrophils are the first cells to migrate to the site of infection; these cells possess a highly bactericidal capacity through the release of proteolytic enzymes, antimicrobial peptides, and reactive oxygen species (ROS). In addition, the ability that has been recently demonstrated, in fish neutrophils is that of releasing neutrophil extracellular traps (NET), which are complex structures consisting of DNA, histones, and proteins from granules. These structures are responsible for trapping and extracellular killing of bacteria, fungi, parasites, and also for inactivating viruses [19]. Thus, it has been demonstrated that NETs production can be ROS-dependent or -independent. Finally, fish eosinophils, as in mammals, release cytoplasmic granules against extracellular parasites [20, 21].

### 1.3. Adaptive Immunity

Adaptive immunity mechanisms in fishes play a vital role in protection against recurrent infections through the generation of cellular and humoral mechanisms, which generate immunological memory, mediated by T- and B-lymphocytes and antibodies [22]. Fishes are the first vertebrates in which clonal selection and genetic rearrangement in lymphocyte receptors presents [4].

In fishes, leukocytes have been reported with T-cell activity, similar to that of the T-helper and cytotoxic cells of mammals; also, in some species of fish, some structurally conserved cellular markers are present, including Clusters of differentiation (CD)3^+^ and T-cell receptors (TCR). Furthermore, based on the profile of cytokines, these also possess T-cell subpopulations similar to those reported in mammals [23].

B-cells are characterized by the expression of antigen receptors (B-cell antigen receptors (BCR)) in the membrane. In teleost fish, B-cells activated, plasmablasts, and plasma cells were identified, which were differentiated from each other mainly by their ability in the production of antibodies [24, 25].

Soluble antibodies that have been identified in fishes are primarily (IgM), which are tetrameric and present in high concentrations in plasma and IgD, which, as in mammals, are expressed on the surface of B-lymphocytes. Other antibody isotypes have been identified in fishes, including IgT and IgZ, which are present mainly in the mucosa, such as in intestine, skin, and gills [26, 27]. Innate and adaptive mechanisms in fishes, similar to those occurring in mammals, are regulated and interconnected by cytokines. Of these, interleukin 1*β* (IL-*β*), IL-6 and tumor necrosis factor alpha (TNF-*α*) have been well characterized in these organisms. Other cytokines reported in fish include IL-2, IL-4, IL-8, IL-10, IL-12, and tumor growth factor beta (TGF-*β*) [28–30]. In addition to cytokines, the immune system of teleost fishes is regulated by neuroendocrine interactions, primarily through the hypothalamic-pituitary-interrenal (HPI) axis, because the pronephros, in addition to their roles as lymphoid organs, has important endocrine functions. However, there is also evidence of the effect of hypothalamic-pituitary-thyroid (HPT) and the brain intercommunication-pituitary-gonadal axes on the immune response in teleost fish [31].

## 2. Cholinergic System in Teleost Fish

**Figure 2:**
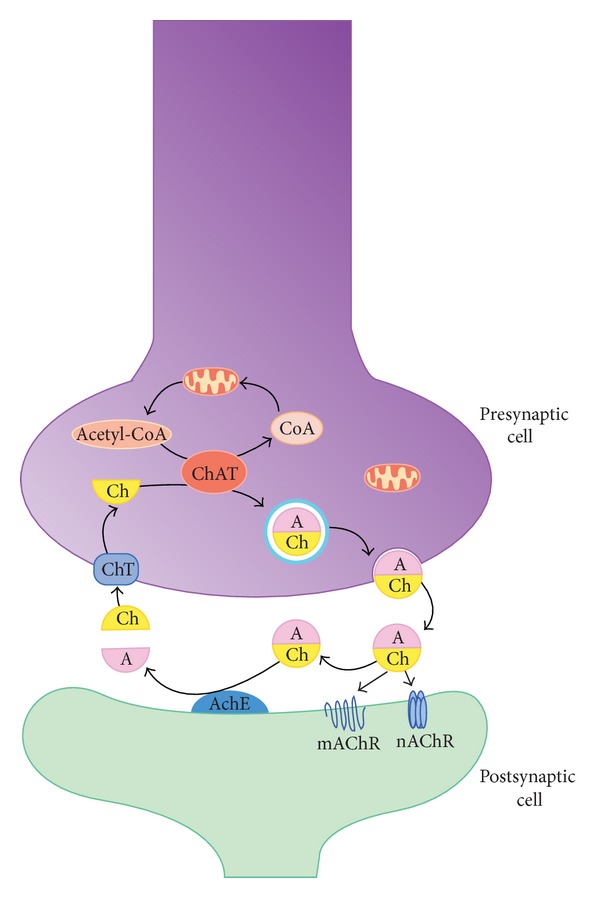
Synthesis of acetylcholine in synapse. A: Acetyl; AChE: acetylcholinesterase; Ch: choline; ChAT: acetylcholine transferase; ChT: choline transporter; CoA: coenzyme A; mAChR: muscarinic receptor; nAChR: nicotinic receptor.

Acetylcholine (ACh) is a neurotransmitter that widely distributed in the central and peripheral nervous systems. It is synthesized from choline and acetyl-Coenzyme A (acetyl-CoA) by the enzyme choline acetyltransferase (ChAT) and then is stored in presynaptic vesicles until the cell is activated (Figure 2). When ACh is released into the nerve synapse, this neurotransmitter binds two distinct receptors on the postsynaptic cell: the ionotropic nicotinic acetylcholine receptor (nAChR) and the metabotropic muscarinic acetylcholine receptor (mAChR), which are bonded to G protein. In the synaptic cleft, ACh is hydrolyzed by the enzyme acetylcholinesterase (AChE) into choline and acetate; approximately 50% of the choline hydrolyzed is recovered by the high-affinity presynaptic transporter, which achieves continuous production and releases neurotransmitters [35].

There are evidences that ACh is expressed in bacteria, algae, protozoa, and primitive plants, suggesting an early onset of ACh in evolution. These cells utilize ACh as a neurotransmitter. In fish, the following are some functions that have been linked with this neurotransmitter: visual response of optical circuits; gustatory information processing during feeding, and the processing of motor information [36, 37].

To understand the teleost cholinergic system, it is relevant to identify some important anatomical data about their sympathetic nervous system (SNS), which appears to have particularly unique evolutionary traits for engaging in sophisticated underwater life. This system in teleosts consists of sympathetic ganglia associated with the corresponding spinal nerves, a pair of sympathetic trunks connecting the sympathetic ganglia and the splanchnic nerves. A unique feature of the teleost SNS is that the sympathetic trunks extend into the cranial region and connect several cranial sympathetic ganglia that are associated with the cranial (trigeminal, facial, glossopharyngeal, and vagal) nerves. Organization of the cranial sympathetic ganglia varies among species [38]. In many teleost species, a pair of celiac ganglia is present at the point where the celiac arteries emerge from the aorta. The postganglionic fibers emerging from the celiac ganglia are distributed to the coelomic organs, along with the celiac artery. The celiac ganglia are connected to the sympathetic trunk via the splanchnic nerves. 

In mammals, the sympathetic preganglionic neurons (SPN) are clustered in discrete nuclear columns. Developmental studies have demonstrated that SPN, together with the somatic motor neurons, differentiate from a common primitive motor column [39]. In teleosts, the majority of SPN in the sympathetic ganglia appears to be adrenergic; however, a population of ganglion cells (<1%) in the cranial sympathetic ganglia are positive for ChAT; thus, these might be cholinergic [40]. On the other hand, AChE-positive neurons are observed in the periaqueductal gray (PAG) (central gray) in some species of teleost fish, and ChAT-positive neurons were not found dorsally to the central canal but scattered in the lateral region of the central gray [38].

In a comparative study of four fish types, it was observed that distribution of the forebrain cholinergic cells is markedly different among species, suggesting that some structures appeared after the cholinergic system, while in the brainstem, cholinergic structures are well preserved during evolution [41]. The developmental pattern of ChAT-positive neurons has been described in the zebrafish; Arenzana et al. (2005) mentioned that during this fish's development, these neurons are detectable in the forebrain and in the mesencephalic tegmentum region, from 60 hpf, while in the optic tectum, the midbrain does not appear until hatching. In the cerebellum, these cells were observed in the isthmic region and medulla oblongata at the end of the embryonic life. Finally, in the spinal cord, motoneurons are detected from 48 hpf [32]. 

**Figure 3:**
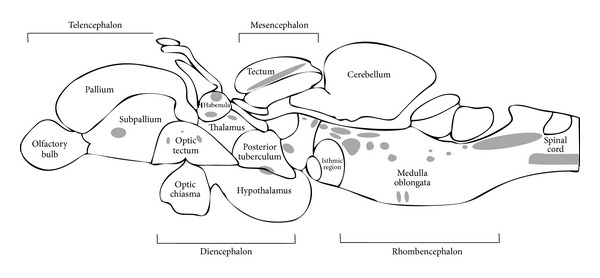
Schematic illustration of location of central nervous cholinergic neuronal populations (gray regions) in adult zebrafish brain (adapted from [32]).

Several studies have shown that the organization of the cholinergic system in the central nervous system (CNS) is similar among vertebrates; in fishes, however, there is greater variability [42]. Mueller et al. (2004) note the presence of ChAT-positive neuronal cells in different regions of the zebrafish brain, for example, in the telencephalon, preoptic region, diencephalon, mesencephalon, isthmic region, and rhombencephalon [43] (Figure 3).

The nicotinic acetylcholine receptor (AChR) is an integral protein of the postsynaptic membrane that has been studied since the 1970s, first in fishes (as in *Electrophorus* spp. and *Torpedo* spp.) and later in mammals (from mammalian muscle). mAChR are related to neurotransmission, neuromodulation, and olfactory mechanisms, while nAChR are involved in glutamate release and memory construction; both receptors have been characterized in zebrafish [44]. Steele et al. (2007) [45] suggest a role for the mAChR in regulating the heart rate under hypoxia in zebrafish larvae, while the function of the nAChR receptor was elucidated by exposing zebrafish to low doses of nicotine, causing effects on the memory of the fish, in addition to anxiolytic effects, as evidenced by swimming upright [46, 47].

Regarding the characterization of the AChE enzyme, this has been identified in brain tissue of various tropical fish, such as pirarucu (*Arapaima gigas*), cobia (*Rachycentron canadum*), and Nile tilapia (*Oreochromis niloticus*) [48]. Employing immunohistochemical techniques, Clemente et al. (2004) observed AChE-positive neurons in the olfactory bulb and the telencephalon and the diencephalon region remains the least dense in AChE-positive neurons; these were more abundant in the isthmic region and in medulla oblongata subdivisions [37].

## 3. Cholinergic Influence on the Immune System in Teleost Fish

Currently, the influence of the nervous system on the immune system cells is clear. Thus, there is abundant evidence of the effect of catecholamine, cortisol, and opioids, even serotonin, on the immune response in teleost fish. However, research focused on the study of the effect of the cholinergic system on the immune response of these organisms is very limited [49]. Classical cholinesterase enzymes such as AChE and butyrylcholinesterase (BChE) are sensitive to other neurotransmitters such as serotonin; thus, it could well represent an interface for a crosstalk between these neurotransmitter systems [50, 51]. 

The two types of cholinesterases (ChE) are present in vertebrates; AChE and BChE exhibit an aryl acylamidase activity (AAA), which is effectively inhibited by cholinergic and serotonergic agents (ACh, specific anticholinesterase drugs and serotonin) [50, 51]. Because the serotoninergic system is involved in pathologies such as anxiety and depression, which in turn influences their immunological responses and cholinergic and that serotonergic drugs sensitively inhibit AAA activity, this could represent a point of crosstalk between the cholinergic and serotoninergic systems. However, due to the complexity of these systems and the lack of precise knowledge that continues to prevail with respect to the brain activity of BChE and also concerning the BChE gene itself that appears to have been lost in some fish lineages [52]. This is why the study of the effect of cholinergic components on neuroimmunomodulation is complex; therefore, addressing a more profound analysis of the relationship between these two systems merits a separate discussion [53]. 

Although the mechanisms of neuromodulation for the cholinergic system in mammals have been extensively elucidated, investigations of this intercommunication in fish are scarce. In this regard, one of the first reports was conducted by Flory (1990) and Flory and Bayne (1991), who demonstrated, in rainbow trout (*O. mykiss*), that carbachol (a cholinergic agonist) significantly increases the number of antibody-producing cells and the concentration of ROS in leukocytes [54, 55].

Related studies on this fish's spleen structure have shown that this lymphoid organ presents an autonomic innervation. Work on the atlantic cod (*Gadus morhua*) has revealed that the teleost spleen receives cholinergic nervous input through a branch of the anterior splenic nerve [56]. Experiments in tench fish or dog fish (*Tinca tinca*) and atlantic cod have shown that exposure to ACh induces a significant reduction of splenic tissue, while exposure to atropine reversed this effect [57].

**Figure 4:**
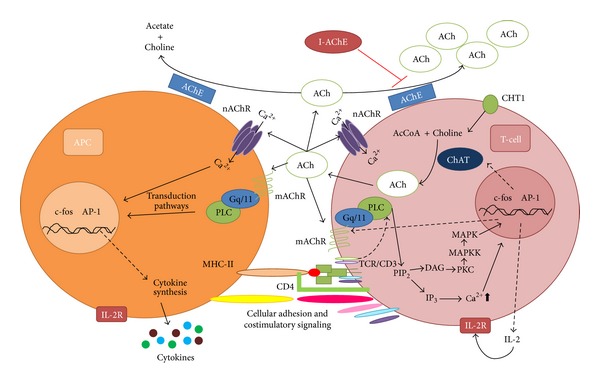
Cholinergic system in cells of the immune system and the effect of acetylcholinesterase inhibitors in mammals. AcCoA: acetyl coenzyme; Ach: acetylcholine; AChE: acetylcholinesterase; AP1: activator protein 1; APC: antigen presenting cell; ChAT: choline acetyltransferase; CHT1: high-affinity choline transporter; DAG: diacyl glycerol; I-AChE: acetylcholinesterase inhibitor; IL-2: interleukin 2; IP_3_: inositol-1,4,5-trisphosphate; mAChR: muscarinic ACh receptor; MAPK: mitogen activated protein kinase; MAPKK: MAP kinase kinase; MHC II: major histocompatibility complex class II; nAChR: nicotinic ACh receptor; PIP_2_: phosphatidylinositol 4,5-bisphosphate; PKC: protein kinase C; PLC: phospholipase C; TCR: T-cell receptor (adapted from [33, 34]).

Moreover, numerous studies have shown that anticholinergic substances (AChE activity inhibitors), such as organophosphorus pesticides (POF), are able to modulate the immune response of fish, leading in the majority of cases to its immunosuppression [33, 34, 58]. Notwithstanding this, the mechanism of immunotoxicity of these compounds remains unclear. Studies by our research group have shown that exposure, *in vivo*, of Nile tilapia (*O. niloticus*) to diazinon (an AChE inhibitor) reduces the proliferative capacity of splenocytes and increases ACh concentration in the spleen, while *in vitro* exposure to this pesticide or to diazoxon (main metabolite of diazinon) does not affect lymphoproliferation. However, lymphocytes exposed to ACh exhibited reduced lymphocyte proliferation [59, 60], suggesting a possible effect of the POF immunotoxicant through alterations in neuroimmunomodulation through cholinergic pathways (Figure 4).

### 3.1. Nonneuronal Cholinergic System and Lymphocytes

ACh is a major neurotransmitter and its presence has been demonstrated not only in neuronal tissue, but also in prokaryotic and eukaryotic cells, from bacteria to mammalian cells, suggesting the presence of this molecule along evolution [36].

In mammals, the presence has been demonstrated of ACh in extraneuronal tissue, including gastrointestinal epithelium, respiratory, urogenital, placental, and vascular endothelial cells and lymphocytes [61]. ChAT enzyme is constitutively expressed in virtually all cells. In nonneuronal cells, ACh is synthesized and released continuously, in small quantities, into the extracellular environment to maintain cellular homeostasis and to regulate basic cellular functions such as mitosis, differentiation, cytoskeleton organization, and cellular interactions [35].

In terms of immune system cells, it has been demonstrated that these possess, in their membrane, muscarinic (mAChR) and nicotinic (nAChR) receptors, through which it regulates their function [61–64]. Furthermore, ChAT enzyme expression in CD4^+^ and CD8^+^ T-cells has been confirmed, suggesting that lymphocytes possess all of the necessary biochemical machinery to produce this neurotransmitter, thereby regulating their functions in an autocrine manner [65]. Furthermore, *in vitro* studies with human mononuclear cells have reported the presence of concentrations of ACh in 0.3 pmol/10^6^ cells, with a synthesis capacity of 2.90 ± 0.84 nmol/mg protein/h [66].

Experimental data obtained by means of *in vitro* models and in the absence of neuronal innervation have shown ChAT production in B cells, macrophages, and dendritic cells from mouse; production of this enzyme appears to be upregulated by TLR activation, a pathway through MyD-88 [65]. Moreover, Neumann et al. in 2007 [66] showed, in human leukocytes, that antagonists of nicotinic and muscarinic receptors (tubocumarin and atropine, resp.) significantly decreased the phagocytic functions of granulocytes, but did not change the migration of these cells, whereas in Jurkat cells (the human helper T-lymphocyte leukemic line), exposure to oxotremorine-M (Oxo-M), a cholinergic agonist, significantly increases the synthesis of IL-2, which could be related with transcriptional factor activator protein-1 (AP-1) and mitogen-activated protein kinases (MAPK) [67], while in experiments in MOLT-3 cells (the human T-cell leukemia line), the involvement has been suggested of the protein kinase C (PKC) signaling pathway-MAPK, cyclic adenosine 3′,5′-monophosphate (cAMP), and calcineurin in the synthesis of ACh [64] (Figure 4).

In general, according to the data obtained in mammalian models, it has been proposed that cholinergic activity increases as a result of direct contact between TCR/CD3 molecules, CD4 and CD8 coreceptors, and other accessory molecules [63]. However, cholinergic component data and extraneuronal cholinergic neuroimmunomodulation mechanisms in fish are scarce.

## 4. The Cholinergic System in Fish: Another Approach in Biomedicine

The cholinergic system, in addition to its exerting a significant influence on the functioning of the immune system of vertebrates, is also essential for homeostasis of the organism. Cholinergic components are related to physiological changes caused by insecticides, poisons, and chemical weapons, as well as by human degenerative diseases such as myasthenia gravis and Alzheimer's disease (AD) [68].

Therefore, besides the study of cholinergic influence on the immune system of fishes, there is now a growing interest in developing new biological models that permit the study of neuromodulation. Among the species of fish that have been employed in this aspect, we find Pacific electric ray (*Torpedo californica*), eel (*Electrophorus electricus*), goldfish (*Carassius auratus*), Nile tilapia (*O. niloticus*), and zebrafish (*Danio rerio*) [69–72].

Myasthenia gravis is an autoimmune disease in which autoantibodies are generated against a cholinergic receptor. Research utilizing fishes as a model have been prominent in the study of this disease. Some species, such as *T. californica*, have electric organs whose function is dependent on the cholinergic system; thus, these organisms are a rich source of AChR, molecules that have been investigated to determine the epitopes related to the development of this disease [73].

Another disease in which the use of fish is proposed as a study model is AD, most common form of dementia and which is characterized mainly by massive neuronal loss and impaired synaptic processes localized in the cerebral cortex, particularly in the frontal and temporal lobes and the hippocampus. AD is related to cholinergic system dysfunctions, such as the loss of cholinergic neurons in the basal forebrain and the hippocampus. In this regard, the effect of various cholinergic drugs has been evaluated in zebrafish and it has been reported that scopolamine (a cholinergic muscarinic receptor antagonist) impairs both the acquisition of the passive avoidance response and the retention of the learned response and that physostigmine (an acetylcholinesterase inhibitor that blocks the breakdown of the ACh released at the synaptic site) rescues the amnesic effects of scopolamine. Altogether, these findings could facilitate the use of the zebrafish as a model for the study of cholinergic mechanisms underlying learning and memory [74]. Moreover, studies related to the development of memory have been conducted in zebrafish; the data indicate that nicotine affects the memory of these organisms, similar to what has been reported in mammalian models [75]. Thus, this fish can be used to help understand the molecular mechanisms of the cholinergic system's influence on cognitive functions.

Fishes also have been used to evaluate the effects of neurotoxins, such as the case of anatoxin-a, a nicotinic agonist produced by cyanobacteria that blocks cholinergic neurotransmission to compete for the ACh receptor. This toxin can cause death in humans and other animals. Studies on rainbow trout (*O. mykiss*) indicate that exposure to this toxin induces increased AChE and lactate dehydrogenase activity, suggesting that this neurotoxin induces motor impairments and increases the metabolic demand of exposed organisms [76].

The effect of anticholinesterase pesticides has also been extensively studied in fishes. Carbofuran is a pesticide of the carbamates group that is highly toxic to mammals. In humans, this substance causes salivation, abdominal pain, chest tightness, dizziness, vomiting, and seizures. Studies on the common carp (*Cyprinus carpio*) indicate that this pesticide reduces the hatching rate and also induces body deformities, eye pigmentation, pericardial sac enlargement, and changes in fish behavior [77]. Also, carbofuran induces neuroendocrine dysfunctions in spotted snakehead fish (*Channa punctatus*) and abnormalities in the thyroid gland, possibly through an alteration of the hypothalamic-pituitary-thyroid (HPT) axis of the fish [78]. Moreover, immunotoxicity studies of this type of pesticides have shown that teleost fish comprise an excellent model for both basic research and ecotoxicology studies [79]. Studies carried out in our research group have shown that exposure to diazinon induced in Nile tilapia (*O. niloticus*) increased the RB of phagocytic cells and serum IgM concentration, but this pesticide caused a decrease in the proliferative and phagocytic capacity of leukocytes [59, 80], while chlorpyrifos, another anticholinesterase inhibitor, induced phagocytic index reduction in this fish [81]. Thus, the immunotoxic effects of anticholinesterase pesticides in vertebrates may be associated with alterations in neuronal cholinergic and extraneuronal cholinergic pathways.

## 5. Concluding Remarks

Studies on the communication between the cholinergic and the immune systems in fish are scarce. However, this type of study could generate relevant data to contribute to the understanding of this bidirectional communication that once a full understanding of neuroendocrine control in fish has been achieved, could approach the study of bidirectional communication in evolutive terms, in addition to understanding the importance of the nonneuronal cholinergic system in nonmammalians models. These approaches certainly guarantee a better understanding of basic aspects and eventually allow the proposal of pharmacological alternatives in clinical medicine. Thus, the use of fish as a biomedical research model could contribute to a better understanding of neuroimmunomodulation mechanisms in vertebrates.
